# Transcriptomics comparison reveals the diversity of ethylene and methyl-jasmonate in roles of TIA metabolism in *Catharanthus roseus*

**DOI:** 10.1186/s12864-018-4879-3

**Published:** 2018-07-02

**Authors:** Ya-jie Pan, Ying-chao Lin, Bo-fan Yu, Yuan-gang Zu, Fang Yu, Zhong-Hua Tang

**Affiliations:** 1 The Key Laboratory of Plant Ecology, Northeast Forestry University, Harbin, 150040 China; 2 Guizhou Academy of Tobacco Research, Guiyang, 550081 China; 3 School of Biological Engineering, Dalian Polytechnic University, Dalian, 116034 China

**Keywords:** ABC transporter, *Catharanthus roseus*, Ethylene, Methyl-jasmonate, Next-generation sequencing, Terpenoid indole alkaloids

## Abstract

**Background:**

The medicinal plant, *Catharanthus roseus* (*C. roseus*), accumulates a wide range of terpenoid indole alkaloids (TIAs). Ethylene (ET) and methyl-jasmonate (MeJA) were previously reported as effective elicitors for the production of various valuable secondary metabolites of *C. roseus*, while a few ET or MeJA induced transcriptomic research is yet reported on this species. In this study, the de-novo transcriptome assembly of *C. roseus* is performed by using the next-generation sequencing technology.

**Results:**

The result shows that phenolic biosynthesis genes respond specifically to ET in leaves, monoterpenoid biosynthesis genes respond specifically to MeJA in roots. By screening the database, 23 ATP-binding cassette (ABC) transporter partial sequences are identified in *C. roseus*. On this basis, more than 80 key genes that encode key enzymes (namely TIA pathway, transcriptional factor (TF) and candidate ABC transporter) of alkaloid synthesis in TIA biosynthetic pathways are chosen to explore the integrative responses to ET and MeJA at the transcriptional level. Our data indicated that TIA accumulation is strictly regulated by the TF ethylene responsive factor (ERF) and bHLH iridoid synthesis 1 (BIS1). The heatmap, combined with principal component analysis (PCA) of *C. roseus,* shows that *ERF* co-expression with *ABC2* and *ABC8* specific expression in roots affect the root-specific accumulation of vinblastine in *C. roseus*. On the contrast, *BIS1* activities follow a similar pattern of *ABC3* and *CrTPT2* specific expression in leaves, which affects the leaf-specific accumulation of vindoline in *C. roseus*.

**Conclusions:**

Results presented above illustrate that ethylene has a stronger effect than MeJA on TIA induction at both transcriptional and metabolite level. Furthermore, meta-analysis reveals that ERF and BIS1 form a positive feedback loop connecting two ABC transporters respectively and are actively involved in TIAs responding to ET and MeJA in *C. roseus*.

**Electronic supplementary material:**

The online version of this article (10.1186/s12864-018-4879-3) contains supplementary material, which is available to authorized users.

## Background

Alkaloids are diverse groups of low-molecular-weight nitrogen-containing compounds found in about 20% of plant species and approximate 12,000 alkaloids have been elucidated for their chemical structures [1]. Due to their strong and divergent biological activities, many alkaloids have been extensively applied to clinical therapy [2]. *Catharanthus roseus*, a Terpenoid Indole Alkaloids (TIAs) producing plant, is the sole source of the anti-cancer compounds, namely vinblastine and vincristine [3–10]. However, the accumulation of these anti-cancer components is exceedingly limited, which hardly meet the demand worldwide. Studies on the elucidation of the biosynthesis pathway and pathway regulations for these compounds production in *Catharanthus roseus* can be beneficial for promoting the production by metabolic engineering manipulation.

During the past several decades, efforts were made to the discovery of TIAs biosynthesis genes, regulation on TIAs biosynthesis pathway and elucidation of pathway intermediates transport [8, 11–15]. Although great progress was achieved, there are still many undefined pathway genes, pathway regulators and TIA transporters that require further identification for a better understanding of the production of anti-cancer drug components by *C. roseus*.

Next-generation sequencing (NGS) technologies provide a rapid, cost-efficient way for analyzing genome and transcriptome in non-model species. Recently, many transcriptomic types of research over *C. roseus* were carried out, which dramatically boosted the discovery of TIAs biosynthesis genes and pathway elucidation [2, 11, 14, 16]. In 2015, Kellner et al. generated a genome assembly for *C.roseus* that greatly promoted the discovery of monoterpenoid indole alkaloid (MIA) biosynthesis and identified many putative missing pathway genes, transcription factors (TFs) and intermediates transporters [17].

According to the previous research, it is showed that the expression of pathway genes for TIAs biosynthesis is strictly regulated by development-, tissue- and cell-specific controls in response to various environmental stimuli, both biotic and abiotic, in *C. roseus* [12, 17–20]. At the transcription level, the biosynthesis of these TIAs is also extensively regulated by many transcriptional activators and repressors. Apart from pathway genes and transcription regulators, transporters involved in alkaloid translocation also attracted many concerns for TIAs production engineering by manipulating intermediates transport at both intra- and inter- cellular levels [8, 14, 16, 21].

Biosynthetic pathway of TIAs exists in different types of membranes both inside and between cells in *C. roseus*. The ATP-binding cassette (ABC) proteins can be classified as a large, widespread family; a big part of ABC family is membrane-associated primary transporters. According to past studies, it is discovered that ABC transporters in plant participate in various vital physiological processes [14, 22]. It is widely perceived that ABC transporters can bring out vincristine and vinblastine from human cancer cells. Furthermore, ABC transporters are recently discovered to participate in the process of transporting different monoterpenoid alkaloids in *C. roseus* cells [14]. Our previous works showed that there are at least 12 steps of intermediates transport in the TIAs biosynthesis pathway and an ABC transporter, *CrTPT2*, is identified as a specific MIA transporter controlling the secretion of catharanthine from leaf epidermis to leaf surface [14].

In *C. roseus*, two important plant hormones, ethylene (ET) and methyl-jasmonate (MeJA), were confirmed to be involved in regulating TIAs biosynthesis. Many pathway genes and transcriptional regulators were reported to be regulated by these plant hormones, which indicated that *Catharanthus* TIAs biosynthesis was involved in MeJA and ET signaling transduction pathway [18, 23–26]. Investigations of the way these plant hormones regulating TIAs biosynthesis and genes responding to these plant hormones are very helpful for understanding these compounds biosynthesis under different environmental, developmental, and stress conditions.

Recently, the application of NGS technique provided plenty of important information on how MeJA regulated genes expression and of which genes (pathway genes, transcription factors, and putative intermediate transporters) possibly involved in TIAs production [18, 27]. In comparison with most methyl-jasmonate (MeJA) elicited transcriptomic information in studies about *C.roseus*, there was a limitation on deep-investigation of ET-induced TIAs production in this species.

In order to get a comprehensive understanding of the regulation mechanism of ET and MeJA on TIAs production at both transcriptional and metabolic levels, we sequenced transcriptomes of *Catharanthus* plants, treated with ET (30 μM), MeJA (50 μM) and control (CK). By conducting blast and qRT-PCR analysis, 23 candidate ABC family transporter genes were screened out that the expression levels are distinct from each other in response to ET and MeJA. Afterward, more than 80 key genes encoding key enzymes (TIA pathway, transcriptional factor (TF), and candidate ABC transporter) of alkaloid synthesis in TIA biosynthetic pathways were chosen to demonstrate that the dose-dependent response of TIA production to MeJA and ET was correlated with the levels of these TFs and transporters. The integration of these genes expression and TIA profiles suggested a mechanism by which TIA production was controlled at the transcriptional level with MeJA and ET dosage.

## Results

### De novo assembly and quantitative assessment of Illumina sequence

cDNA libraries from *Catharanthus* seedlings with 4 h treatment of ET (30 μM) and MeJA (50 μM) along with control (CK) were prepared for high throughput sequencing by Illumina Hiseq 2000 system. Three biological repeats were prepared for each condition. 32,891,528,340 nt bases were generated totally. After removing the adapter sequences, empty reads and low-quality sequences, 38,018,320 (CK1), 43,830,696 (CK2), and 40,406,978 (CK3) clean reads from CK, 40,601,918 (ET1), 44,225,374 (ET2), and 40,215,966 (ET3) clean reads from ET, and 40,689,468 (MJ1), 40,044,736 (MJ2), and 37,427,970 (MJ3) clean reads from MeJA were obtained. An overview of sequencing and assembled results have been summarized in Additional file 1: Table S1. We then search unigene sequences against protein databases (Nr, SwissProt, KEGG, COG) using blastx (e-value < 0.00001). Protein function information can be predicted from annotation of the most similar protein in those databases (Additional file 2: Table S2).

### Differential gene expression

**Fig. 1:**
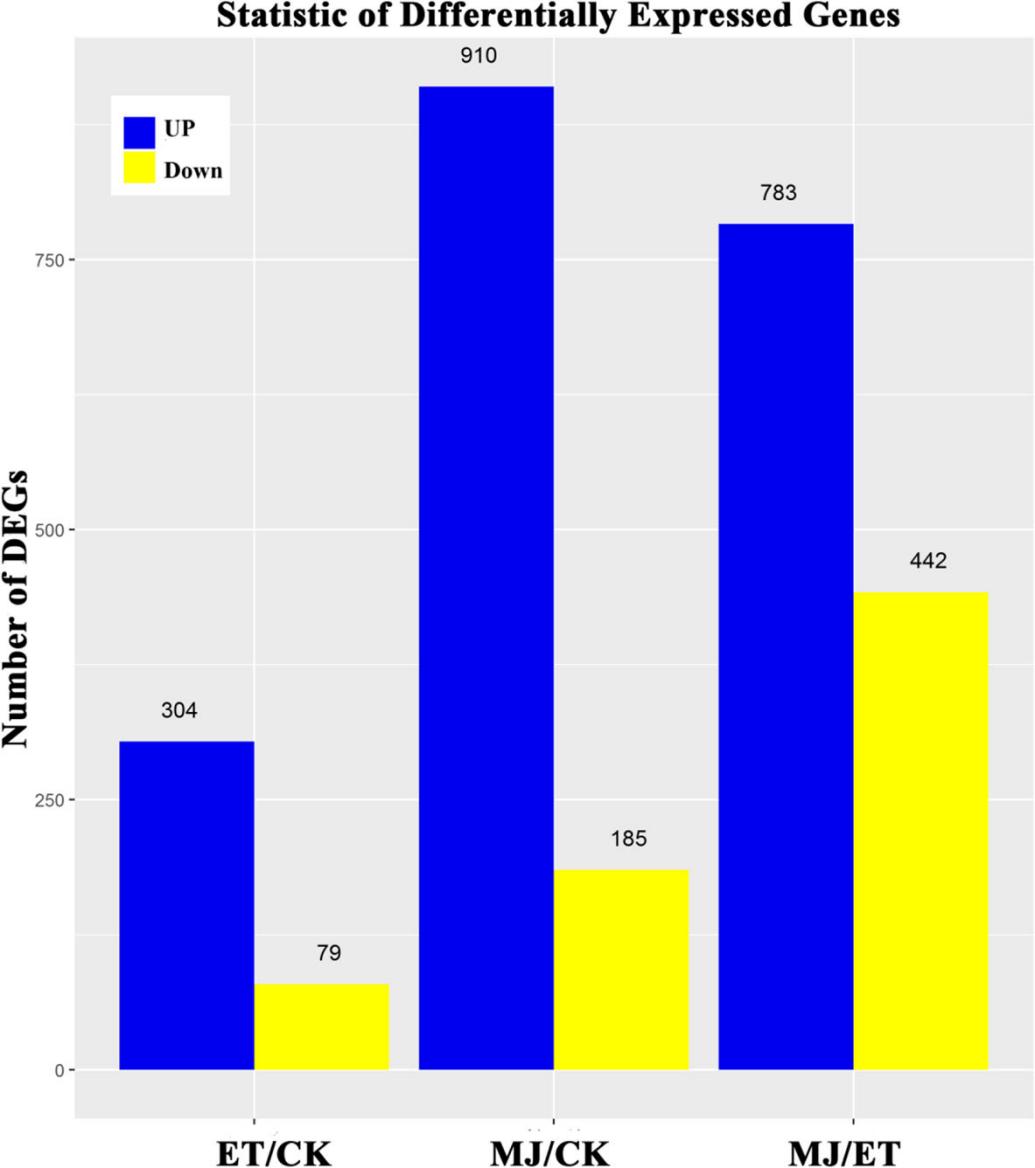
Differential expression analysis of *C. roseus* transcriptome. Statistical chart of DEGs of transcriptomes in response to hormone stress. Compared with the transcriptional level of control (CK, non-treatment), ET and MeJA, 304, 910, and 783 genes were up-regulated, and 79, 185, and 442 genes were down-regulated, respectively. The number of up-regulated genes was shown in blue, while down-regulated genes were shown in yellow

The differentially expressed genes (DEGs) were defined by cuffdiff as those with a false discovery rate (FDR) ≤ 0.001 and fold change equal or larger than 2, as well as requiring Fragment Per Kilobase of exon model per Million mapped reads (FPKMs) > 2. Based on this criterion, we identified 304 (ET vs CK), 910 (MeJA vs CK), and 783 (MeJA vs ET) up-regulated and 79 (ET vs CK), 185 (MeJA vs CK), and 442 (MeJA vs ET) down-regulated genes, respectively (Fig. 1, Additional file 3: Table S3). DEGs were identified in ET and MeJA treated sample cDNA libraries in comparison with that in CK sample cDNA library via pairwise comparisons. A total of 383 and 1095 genes were differentially expressed with ET or MeJA treatment, respectively, which indicated that the number of DEGs with MeJA treatment was considerably bigger than that with ET treatment. With Nr annotation, we use Blast2GO program to get GO annotation of Unigenes. Then we use WEGO software to do GO functional classification for all Unigenes [28]. According to GO enrichment, some important metabolic activities occurred in *C. roseus* in response to MeJA and ET. As shown in Additional file 4: Figure S1, treatment with MeJA specifically enhanced nutrient reservoir comparing with ET. In contrast, translation regulator and viral reproduction were significantly enriched in response to ET. Meanwhile, treatment with MeJA also enhanced biological adhesion, death, immune system process, and rhythmic process comparing to the treatment with ET by GO assignment system (Additional file 4: Figure S1, Additional file 5: Table S4).

### Pathway annotation

**Fig. 2:**
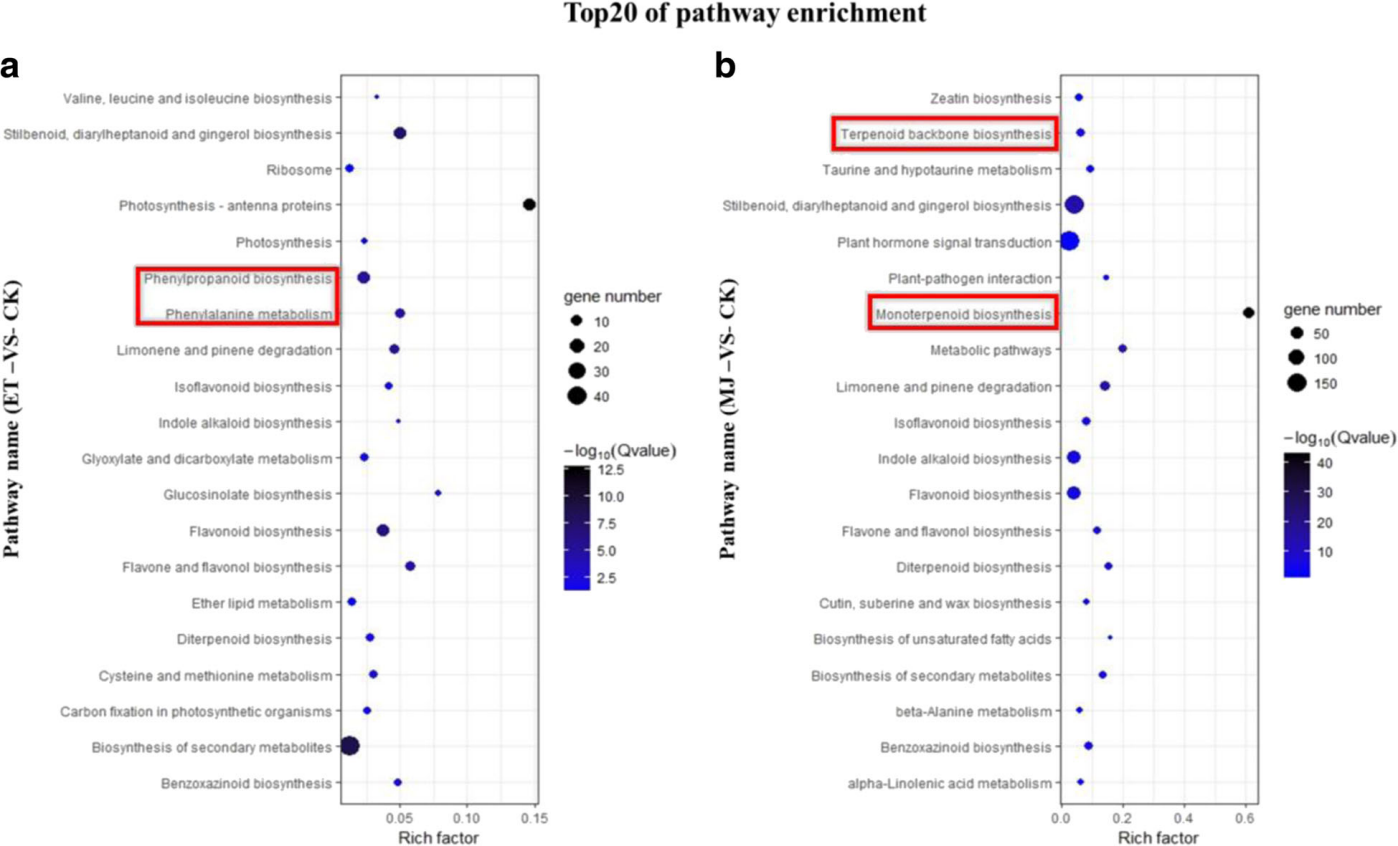
KEGG (Kyoto Encyclopedia of Genes and Genomes) pathway annotation. In total, 30,061 genes (30,061 transcripts) were found to be involved in one of the 401 different KEGG metabolic pathways. We used R Package gplot (https://cran.r-project.org/web/packages/gplots/gplots.pdf) to create the graph. **a** The top 20 statistics of pathway enrichment were represented by comparing ET with control (CK). **b** The top 20 statistics of pathway enrichment were represented by comparing MeJA and control (CK)

The KEGG pathway database records networks of molecular interactions in the cells, and variants of them specific to particular organisms. Pathway-based analysis contributed to further understand biological functions of genes in response to ET or MeJA. In total, 30,061 genes (30,061 transcripts) were found to be involved in one of the 401 different KEGG metabolic pathways in total. In order to compare the specific metabolic pathways in which DEGs participated between ET and MJ treatment, KEGG pathway enrichment analysis was conducted. Comparing Fig. 2a, b, it was found that Phenylpropanoid biosynthesis (9.9%), Phenylalanine metabolism (5.0%) genes responded specifically to ET, Monoterpenoid biosynthesis (9.0%) and Terpenoid backbone biosynthesis (7.0%) genes responded specifically to MeJA (Additional file 6: Table S5). This indicated that the secondary metabolites of *C.roseus* showed special sensitivity when stimulated by exogenous hormones; with respect to transcriptional level, phenolic compounds were more sensitive to ET, while MeJA had a stronger influence over MIA. Among the 64 different KEGG metabolic pathways under ET treatment, 197 genes were selected involving 3 different KEGG metabolic pathways, which were Isoflavonoid biosynthesis (97 genes), Indole alkaloid biosynthesis (41 genes) and Monoterpenoid biosynthesis (59 genes) pathway (Additional file 6: Table S5). Our results showed that 33 of 197 genes in the pathways were up-regulated, while 35 of 197 genes were down-regulated following ET treatment. The results indicated that the ET up-regulated genes mostly associated with two GO terms, metal ion binding (7%) and lyase activity (7%), whereas ET down-regulated genes gathered in the methylation-enzyme-activity group (27.27%, include methylation indole-3-acetate esterase activity, methylation salicylate esterase activity, and methylation jasmonate esterase activity). An interesting finding was that the activity of other hormones metabolic process (include jasmonic acid metabolic process 9.09% and salicylic acid metabolic process 9.09%) was peculiarly decreased (Additional file 7: Figure S2, Additional file 8: Table S6).

### The statistical analysis of TIA pathway genes in response to ET or MeJA by RNA-seq

**Fig. 3:**
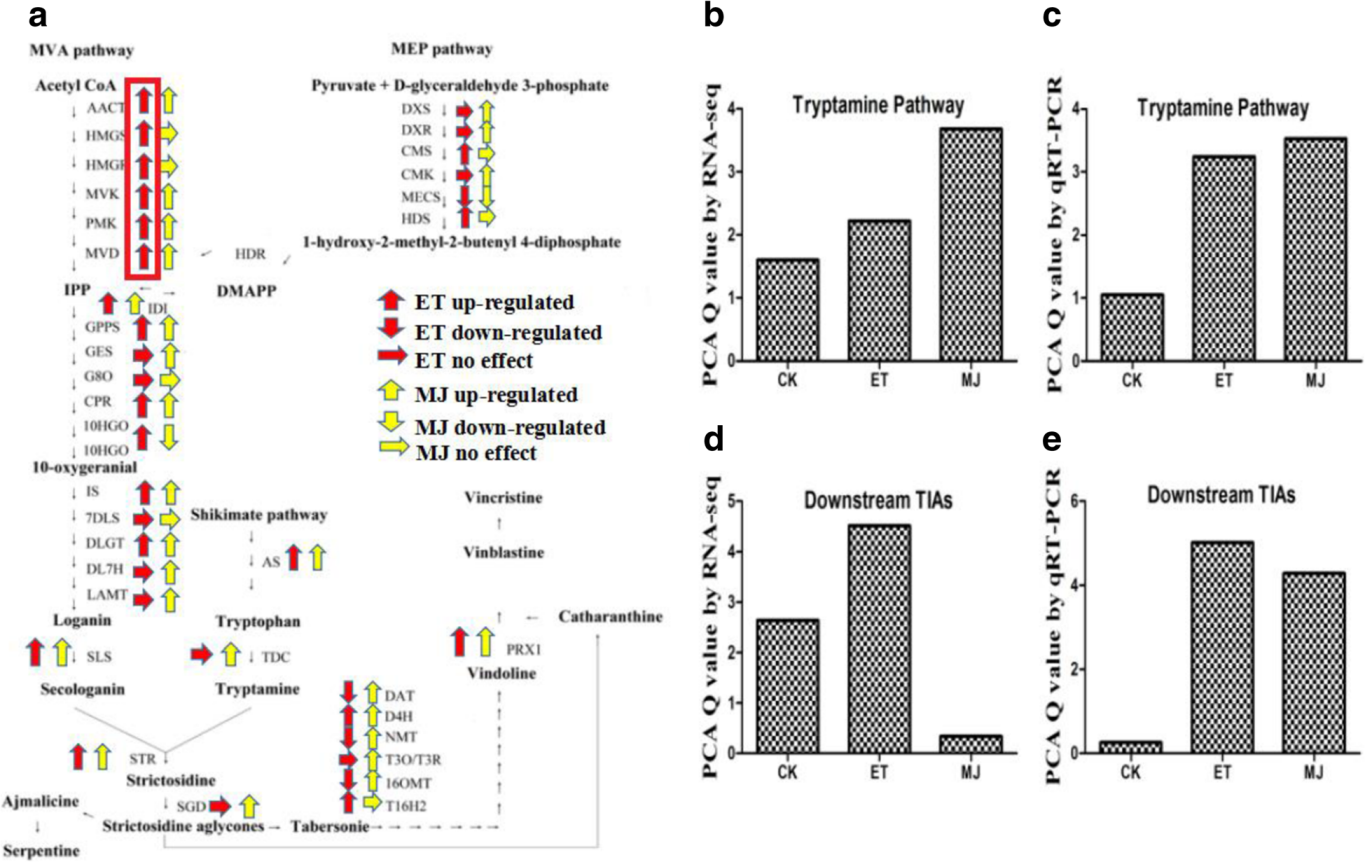
TIA pathway genes expression (by RNA-seq) in response to ET and MeJA treatment. Pathways led to the production of TIAs in *C. roseus*. **a** red arrows denoted ET regulation and yellow arrows denoted MeJA regulation, respectively. **b** Score plot of PCA by RNA-seq (Tryptamine pathway genes). **c** Score plot of PCA by qRT-PCR (Tryptamine pathway genes). **d** Score plot of PCA by RNA-seq (Downstream TIAs genes). **e** Score plot of PCA by qRT-PCR (Downstream TIAs genes)

Under pathway annotation, it was known that secondary metabolic pathways had the largest number of DEGs when treated with ET or MeJA. Based on the references in recent 10 years, 37 key genes were identified after comparing the TIA pathway genes from *C.roseus* with the NCBI database [7, 8, 14]. For further analysis, the expression of all 37 key genes encoding key enzymes of alkaloid synthesis in TIA biosynthetic pathways was chosen by false discovery rate (FDR) threshold with corrected *P*-value < 0.05. By Fragment Per Kilobase of exon model per Million mapped reads (FPKMs) values, no major up-regulated change in expression of *G8O* and *7DLS* genes were observed either under ET or MeJA treatment (Fig. 3a).

Based on our seq data, the expression of *MVK* (3.15 fold) was significantly enhanced after ET treatment. Meanwhile, compared to the control, the expression of *DXS* (4.255 fold), *AS* (3.01 fold), *LAMT* (2.499 fold), and *STR* (2.08 fold) was enhanced greatly, when treatment with MeJA alone (Fig. 3a). We also found that the expression of *NMT* was considerably decreased under the ET treatment, yet the expression of *MECS* was significantly decreased under both the MeJA and ET treatment. Furthermore, as shown in Fig. 3a, all MVA pathway genes (*AACT, HMGS, HMGR, MVK, PMK, and MVD*) were up-regulated in response to ET. The results revealed that the induction effect of ET was stronger over the expression of mevalonate (MVA) pathway genes. In order to investigate the overall expression of TIA pathway genes in response to ET and MeJA treatment, these TIA pathway genes were validated based on different stress stages using quantitative real-time polymerase chain reaction (qRT-PCR) (Additional file 9: Table S7). In principal component analysis (PCA), Q-value was employed to measure the level of gene expression based on the qRT-PCR and RNA-seq data of TIA related genes. According to the data of RNA-seq (Fig. 3b and d) and qRT-PCR (Fig. 3c and e), it was learned that their genes expression in the trend of tryptamine pathway and TIA downstream pathway are different in response to ET and MeJA. Tryptamine pathway genes were more sensitive to MeJA (Fig. 3b and c), whereas TIA downstream pathway genes were more sensitive to ET (Fig. 3d and e).

### Gene ontology (GO) classification of ATP-binding cassette (ABC) family genes

**Fig. 4:**
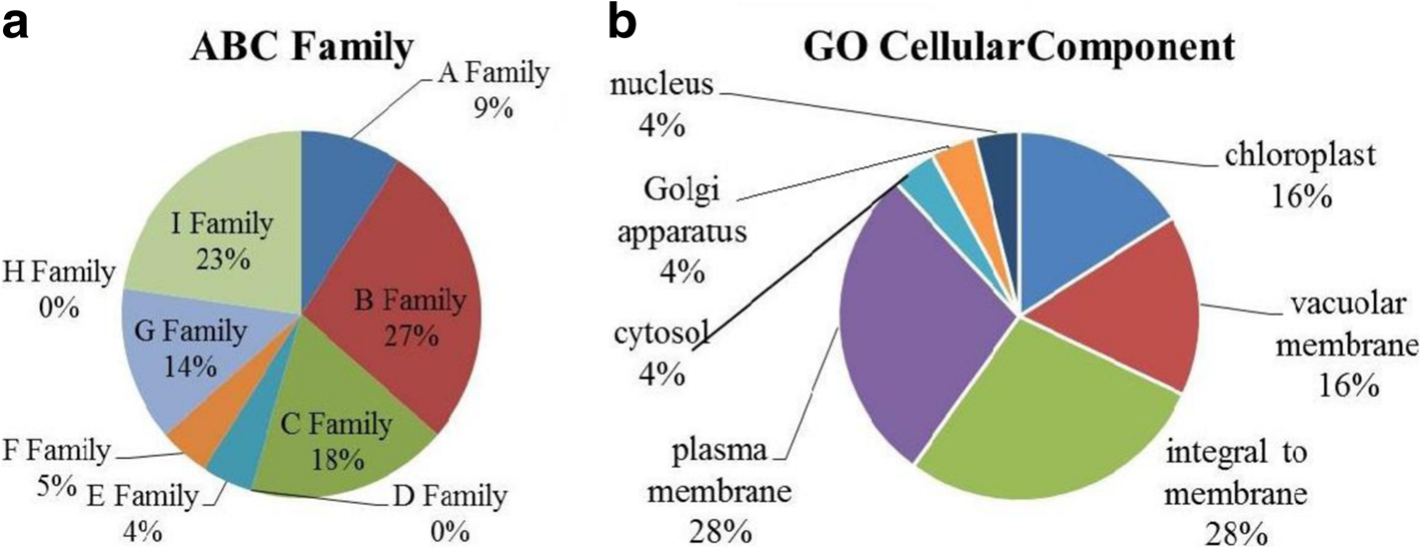
Gene Ontology classification of 23 candidate ABC genes. **a** Putative 23 candidate ABC transporters were initially assigned to the respective subfamilies (A-H, except D and H) based on BLAST analysis. **b** 23 unigenes are summarized into Gene Ontology cellular location categories

ET and MeJA have abundant physiological functions and participate in many vital processes in plant growth and department. How these plant hormones participate in TIA metabolism process is still unknown in *C.roseus*. We know that lots of transport proteins, including ABC families, are involved in the process of TIA transmembrane transport in *C.roseus*. By conducting blast analysis, we collected 339 candidate transporter genes that were annotated as part of ATP-binding cassette (ABC) family (Additional file 10: Table S8). On this basis, the orthologs of *C. roseus* which have a significant differential expression of 50 candidate transporter genes were selected for further analysis using qRT-PCR. Among them, 23 transporters were screened out that their expression levels are distinct from each other in response to ET and MeJA. As is shown in the results, the 23 candidate genes perfectly reflected to categorize into 339 candidate ABC transport families (A-I), among 23 candidate genes the ABC-B family contained the most genes, with the percentage of 27. Nevertheless, genes that belong to ABC-D family and ABC-H family were not detected. ABC-C family was widely studied, to which 18% candidates were classified (Fig. 4a). Meanwhile, the GO assignment system was applied to obtain functional information of the ABC family sequences at the macro level. For the 23 candidate genes after ET and MeJA treatment, the GO terms related to the plasma membrane (28%) and integral to membrane (28%) were significantly enriched (Fig. 4b).

### The heatmap analysis of TIA related genes in response to ET or MeJA by qRT-PCR

**Fig. 5:**
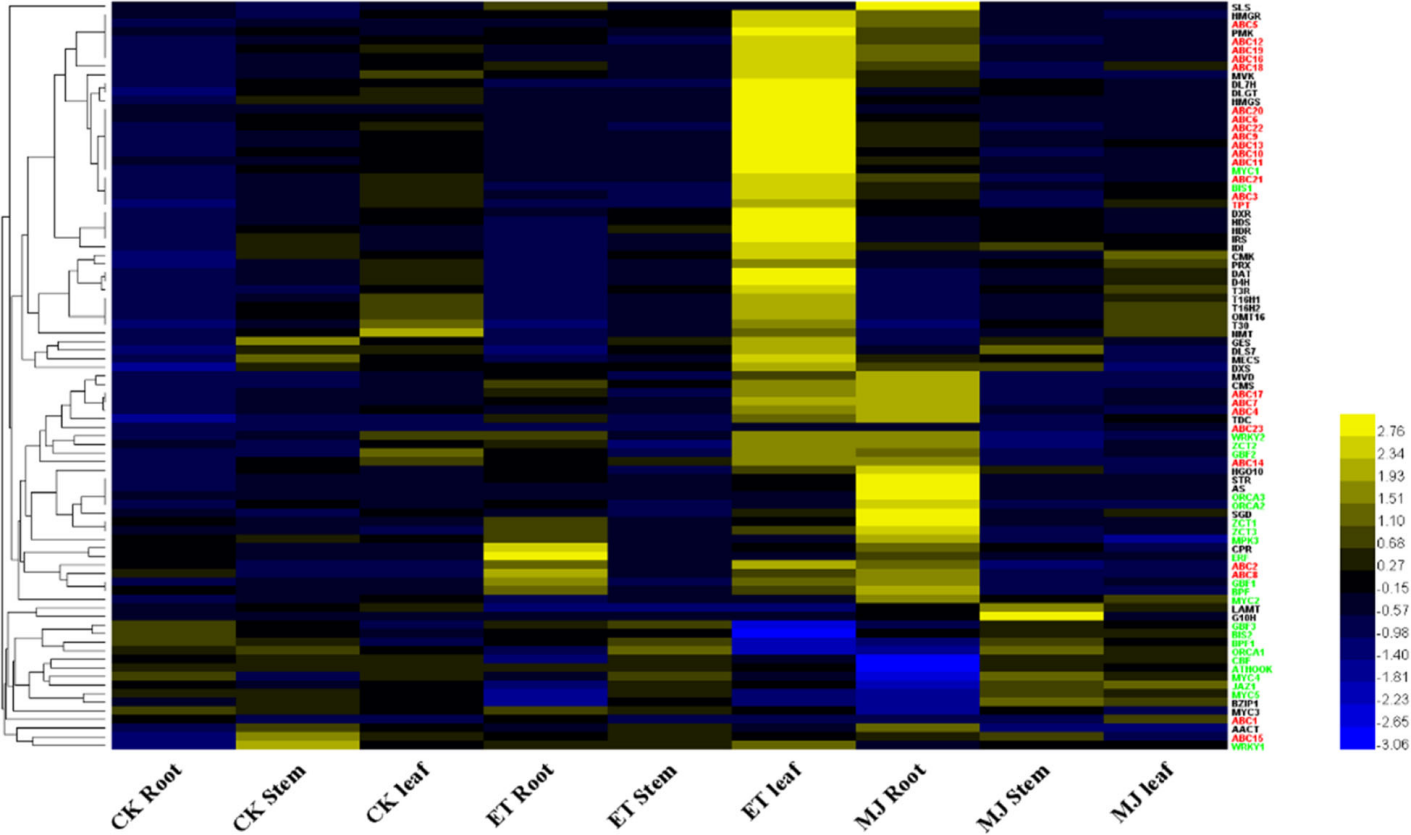
Heatmap analysis of TIA related genes. Heatmap was employed based on the qRT-PCR data of TIA related genes. The qRT-PCR results were analyzed using the comparative Ct method (The *Rps9* gene was used as an internal control). Relative expression of genes in *C.roseus* leaves, roots and stems upon control, 30 μM ET, 50 μM MeJA. Each relative gene expression represents the average of three measurements. The data of qRT-PCR results were analyzed by using the Z-score statistical method. The black word is shown as pathway genes, the red word shown as ABC transporter genes, the green word shown as transcription factor genes

In order to investigate the overall expression of TIA related genes (TIA pathway genes, TFs, ABC transporters) from RNA-seq data, heatmap was employed based on the qRT-PCR data of 85 TIA related genes mentioned above in response to ET or MeJA in different tissues (Fig. 5, Additional file 9: Table S7), the data of qRT-PCR results were analyzed by using “Z-score” statistical method. In statistics, the “Z-score” is the signed number of standard deviations by which the value of an observation or data point is above the mean value of what is being observed or measured. Observed values above the mean have positive standard scores, while values below the mean have negative standard scores [29].

The result showed that TIA-related genes exhibited obvious tissue specificity in response to the treatment with exogenous hormones: ET had a more significant effect on the genes in leaves, whereas the genes in roots were more sensitive towards MeJA. As shown in Fig. 5, ET was the one that induced the genes coding TIA pathway, which only expressed in *C. roseus* leaves. Moreover, there was no obvious effect on the expression of these genes in roots or stems, which reveals different tissue-specific regulation patterns in response to ET (Fig. 5). According to the clustering and heatmap analysis of the above three types of genes (TIA pathway genes, TFs, ABC transporters), it was found that TIA pathway genes expression followed a similar trend to that of transporter and TF genes, such as the transporter genes of *ABC2, ABC8* were co-expression with pathway gene *CPR* and TF gene *ERF*, in addition, there was a significant co-expression tendency between the transporter gene *ABC15*, pathway gene *AACT,* and TF gene *WRKY1* (Fig. 5).

### PCA analysis of TIA-related genes in response to ET or MeJA by qRT-PCR

**Fig. 6:**
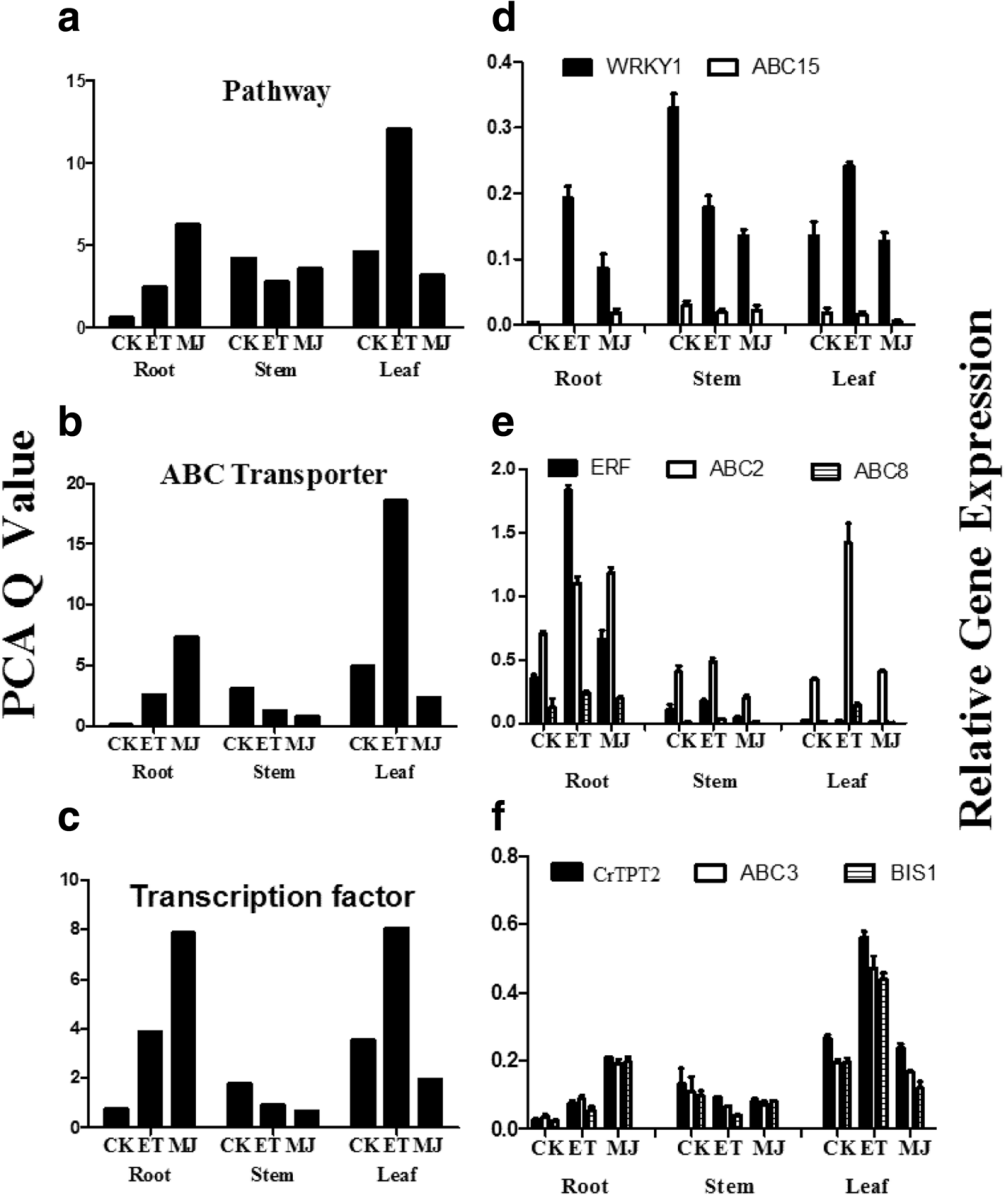
Relative expression levels by qPCR of selected TIA related genes following ethylene application in *C. roseus* leaves, roots, and stems. The qRT-PCR results were analyzed using the comparative Ct method followed by PCA. **a** Score plot of PCA in *C. roseus* (pathway genes). **b** Score plot of PCA in *C. roseus* (ABC transporter genes). **c** Score plot of PCA in *C. roseus* (transcription factor genes). The qRT-PCR results were analyzed using the comparative Ct method (The *Rps9* gene was used as an internal control). Relative expression of genes in *C.roseus* leaves, roots, and stems upon control, 30 μM ET, 50 μM MeJA. Each relative gene expression represents the average of three measurements. **d** Relative expression of *WRKY1* and *ABC15*. **e** Relative expression of *ERF, ABC2,* and *ABC8*. **f** Relative expression of *BIS1, CrTPT2,* and *ABC3*

The spatio-temporal transcriptional regulation of metabolic pathways was controlled by a complex network involving many TFs [14]. As a result, related papers and reports were perused and the TFs that the 24 (ORCA1, ORCA2, ORCA3, CrBPF1, CrMYC1, CrMYC2, CrMYC3, CrMYC4, CrMYC5, CrWRKY1, CrWRKY2, CrERF, AT-HOOK, CBF, BIS1, BIS2, ZCT1, ZCT2, ZCT3, JAZ1, BZIP1, GBF1, GBF2 and GBF3) ET or MeJA relied on were identified. Meanwhile, the function of TFs, as well as transporter genes on responding to the exogenous hormones was analyzed. Thus, PCA was employed and the Q-value was determined based on the qRT-PCR data of TIA related genes in response to ET and MeJA (Fig. 6a, b and c, Additinal file 9: Table S7). The outcome of the experiment revealed that the expression of ABC transporter and transcription factor genes followed a similar trend to that of TIA pathway genes in response to exogenous hormones. Our results showed that ET induced the expression of these three types of genes significantly in leaves, whereas MeJA had a stronger effect on them in roots. Exogenous hormones had relatively less impact in stems. Interestingly enough, the expression level of these three types of genes slightly declined after the exogenous hormone treatment in stems. In the light of the comparative study of the results, ET treatment promoted the expression rates of the three kinds of genes by approximately twice the control treatment expression in leaves. Meanwhile, MeJA treatment increased their expression by around five-fold of control in roots. TIA-related genes in *C. roseus* were possibly more sensitive to ET induction in leaves, whereas these genes were more sensitive to MeJA in roots.

Meanwhile, 24 TFs, 24 ABC transporter genes which were involved in regulating TIAs biosynthesis in *C. roseus* were selected. As is shown in Fig. 6d, *CrWRKY1* expression followed a similar trend to that of *ABC15*. The gene of *ERF* co-expressed with *ABC2* and *ABC8* specifically responded to ET in roots (Fig. 6e). On the contrast, the gene of *CrTPT2* co-expressed with *ABC3* and *BIS1*, it specifically responded to ET in leaves (Fig. 6f). To sum up, the effect of ET and MeJA had stronger induction effect on the expression of TIA-relative genes compared with control.

### Alkaloid synthesis in response to ET or MeJA treatment

**Fig. 7:**
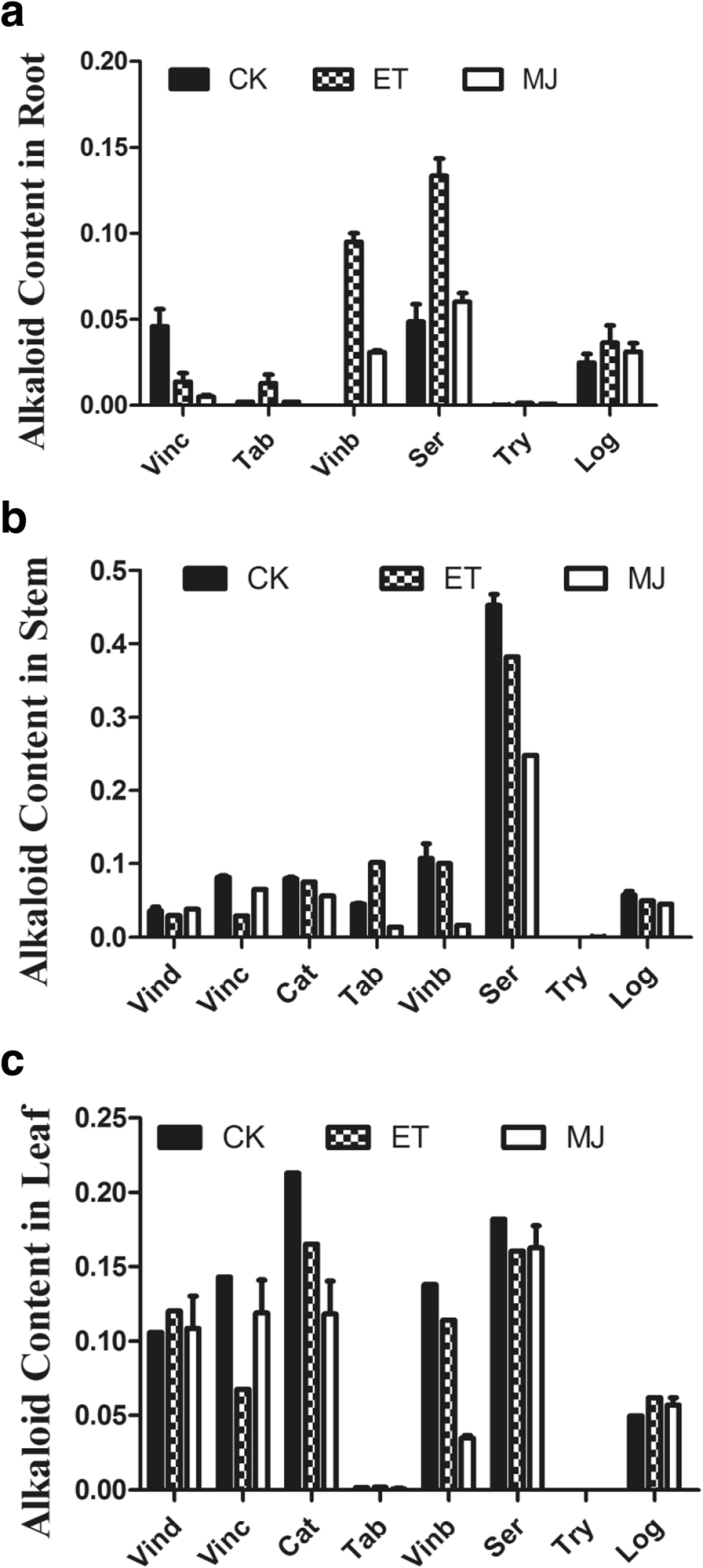
TIAs content (by LC-MS) in response to ET and MeJA treatment. Three-month-old *C. roseus* seedlings were treated with control, ET, and MeJA. TIAs, including secologanin (Sec), tryptophan (Try), strictosidine (Str), tabersonine (Tab), vindoline (Vind), serpentine (Ser), ajmalicine (Ajm), catharanthine (Cat), and vinblastine (Vinb). The average of three measurements

In accordance with a positive response of TIAs biosynthesis genes to ET or MeJA at the transcription level, ET and MeJA also promoted the accumulation of TIAs in *C. roseus*. When the samples were treated with ET, there were no changes observed in stems or leaves except in roots. TIAs, such as serpentine, loganin, vinblastine and tabersonine, accumulated significantly in roots, among which the effect of vinblastine on hormones was most remarkable. A similar pattern was also observed in the response of serpentine to exogenous hormone treatment. Fig. 7a, b and c indicate that the overall contents of serpentine retained at a relatively high level in leaves and roots, especially stems, comparing to other TIAs in *C. roseus*. These alkaloids contents reflected the importance of ET in roots, which directly participated in alkaloids biosynthesis.

## Discussions

Plants produce a vast array of specialized MIAs, many of which are used as pharmaceuticals, flavors, fragrances and other high-value fine chemicals. Several recent reports have provided information about the regulation of TFs and transporters by internal and external signals leading to control responses to increase the accumulation of these compounds [13, 14, 18, 30, 31]. Transcriptome analysis for medicinal plants with the advent of the next generation of sequencing (NGS) technologies provides an opportunity to discover genes involved in the pathways that contribute to the synthesis of plant specialized products. Public databases are presently available for *C.roseus* [14, 18], but no ET elicited transcriptomic information was previously reported on this species. It is interesting to verify whether ET plays a more important role in the biosynthesis of MIA compounds [32].

Plants lack a developed and dynamic vascular system, which explains their need for an elaborated transport system to allow a proper distribution of nutrients and signals. ATP-driven transport (ABC) across biological membranes is a key process to translocate solutes from the interior of the cell to the extracellular environment [33]. Hellsberg’s research also presents that transporters are also known to be involved in phytohormone translocation [33]. In this study, the de-novo transcriptome assembly of *C. roseus* was analyzed using NGS technology. After screening the database, 23 ATP-binding cassette (ABC) transporter partial sequences were identified in *C. roseus*. The transcriptome of *C. roseus* was analyzed to investigate the roles of candidate ABC genes involved in TIA synthesis and to understand mechanisms of ET and MeJA response better in *C. roseus*. Our results showed that pathway genes performed a similar expression trend to ABC transporter genes. In the meantime, ABC transporter genes showed significant tissue specificity when responding to exogenous hormones. ET mainly promoted expression of genes in leaves, while MeJA mainly promoted expression of genes in roots. Moreover, as shown in Fig. 5, TF BIS1 clustered with transporter ABC3, ABC21, the relative quantitative expression of TF WRKY2, ZCT2, GBF2 and transporter ABC14 genes had a similar kinetic trend in response to MeJA and ET. Heatmap analysis revealed that these transporter genes may regulate by corresponding TFs. Although the exact roles of these ABC candidate genes remained to be examined, these results provided a platform for further functional analysis of the process of detoxification of TIA membrane transport mechanisms.

**Fig. 8:**
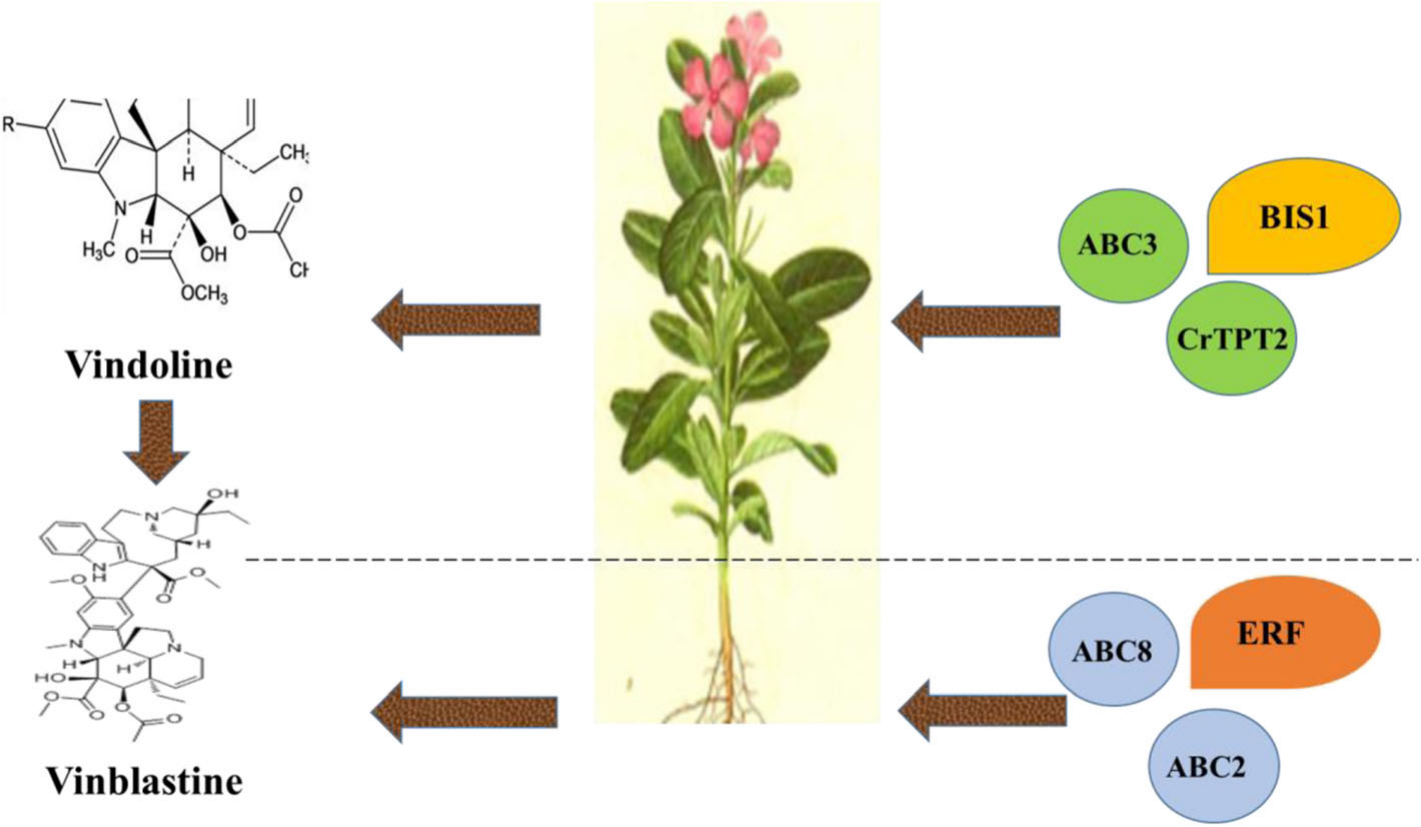
Model for *ERF, BIS1,* and ABC transporter genes regulated TIAs accumulation responding to MeJA and ET signal in *C. roseus*. Transcription factor *ERF* can activate vinblastine via co-expression with *ABC2* and *ABC8* in roots. Also, transcription factor *BIS1* interacts with *CrTPT2* and *ABC3* in a vindoline production pathway in leaves. The TIAs accumulation exclusively regulated by ERF and BIS1 are shown with orange and yellow box, respectively

Biosynthesis of MIAs is achieved by coordinated transcription of MIA pathway genes through TFs and transporters. The TFs and transporters are required for the full spectrum of phytohormone-induced metabolic changes [14, 34]. For instance, *ERF* gene is involved in the regulation of TIA genes in response to ethylene [34]; Moerkercke also found that the bHLH transcription factor BIS1 controls the iridoid branch of the monoterpenoid indole alkaloid pathway in *C.roseus* [33]*.* Wang’s research shows that Mrr2p (multidrug resistance regulator 2) is a novel TF controlling expression of the ABC transporter gene CDR1 and mediating fluconazole resistance [35]. Based on previous studies, several transcriptional regulators and candidate ABC transporters were selected to test whether they formed a complex network to participate in regulating the expression of TIA pathway genes in responding positively to ET or MeJA. Results suggested that the mRNA expression pattern of *ABC8* and *ERF* genes showed distinct co-regulation in response to ET and MeJA treatment, particularly in *C. roseus* roots (Fig. 6e). On the contrast, expression of *BIS1* was significantly related to the expression of *ABC3* and *CrTPT2* mainly in leaves (Fig. 6f). It was shown that ABC transporter genes played a significant role in regulating TIA pathway genes. Previous research has found that *CrTPT2* was expressed predominantly in the epidermis of young leaves. Treatment of 2-wk-old *Catharanthus* seedlings with MeJA increased *CrTPT2* expression four-fold [14]. Further experiments in this study discovered that the expression of *CrTPT2* was 3 times as much in response to ET as MeJA. It appeared that *CrTPT2* is a possible potential candidate for engineering regulation of TIA pathway gene expression and improving alkaloid production when treated with ET. As was indicated by heatmap analysis (Fig. 5), these ABC transporters responded positively to ET and MeJA to involve the expression of enzyme-coding genes, some of which co-expressed with TFs to form a complex network and to participate in the expression of TIA pathway genes. Co-expression of TF and transporter genes significantly enhanced the expression of TIA pathway genes, suggesting that gene expression was coordinated by TF and transporter jointly, thus it promoted TIAs accumulation [14]. A simplified picture of our results of the ET and MeJA induced responses in *C. roseus* (Fig. 8) was developed. In this study, a possible mechanism was presented: it was likely that ERF and BIS1 generated a positive feedback loop to separately connect to two ABC transporter genes to be actively involved in response to ET and MeJA in TIAs in *C. roseus*. For instance, *BIS1* interaction between *ABC3* and *CrTPT2* specific expression in leaves affected the leaf-specific accumulation of vindoline in *C. roseus*; *ERF* interaction between *ABC2* and *ABC8* specific expression in roots affected the root-specific accumulation of vinblastine in *C. roseus*.

The over-expression of *CrWRKY1* in *C. roseus* hairy roots up-regulated several key TIA pathway genes, especially *TDC* and *DXS* [8], which showed that *WRKY1* regulated several TIA upstream pathway genes in response to MeJA. Moreover, the over-expression of *CrWRKY1* also up-regulated the transcriptional repressors *ZCT1, ZCT2,* and *ZCT3*, but repressed the transcriptional activators *ORCA2*, *ORCA3*, and *CrMYC2* [8]. Here the heatmap showed that *WRKY1* was clearly co-expressed with *ABC15* (Fig. 6d). Subsequently, it was deduced that *WRKY* gene exhibited extensive auto-regulation and cross-regulation that facilitated transcriptional reprogramming in a dynamic web with built-in redundancy [8, 36]. Suttipanta’s research also showed that CrWRKY1 in roots possibly played a key role in determining the root-specific accumulation of serpentine in *C. roseus* plants. Findings in this study were consistent with those of Suttipanta’s research, which also indicated that the expression of *CrWRKY1* was more sensitive to ET (Fig. 6d).

Results of differentially expressed genes showed that TIA-related genes were significantly up-regulated under both ET and MeJA treatment. It also illustrated that phenolic biosynthesis genes responded specifically to ET, while monoterpenoid biosynthesis genes responded specifically to MeJA (Fig. 2). The phytohormones ET and MeJA were reported to elicit secondary metabolites such as alkaloids and phenolic compounds regulating plant growth and adaptation [8, 37–40]. ET acted as an intermediate signaling molecule elicitor induced flavonol accumulation alone or interact with auxin [41]. The addition of MeJA to *C.roseus* hairy root cultures increased the yields of ajmalicine, serpentine, lochnericine and hörhammericine [24, 34]. Natural phenolic compounds play an important role in cancer prevention and treatment. Phenolic compounds (include phenolic acids, flavonoids, tannins, stilbenes, curcuminoids, coumarins, lignans, quinones and others), ubiquitous in plants are an essential part of the human diet and are of considerable interest due to their antioxidant properties [18]. It is interesting to verify that whether ET or MeJA played an important role including the biosynthesis of phenolic compounds in the further study.

At both transcriptional and metabolic levels, TIA pathway genes and alkaloids actively responded to ET and MeJA treatments. Results showed that significant accumulation of alkaloids resulted from the application of ET and MeJA, especially in response to ET in the roots (Fig. 7a). The synthesis and accumulation of TIAs were strictly controlled. This study showed that the iridoid (terpenoid) and indole alkaloid pathways were also controlled by posttranscriptional and posttranslational mechanisms. Assembly of TIAs in *C. roseus* was remarkably dynamic, involving at least three cell types in leaves [14]. Rates of biosynthesis, as well as directional transport mechanisms, precisely regulate where, when, and how different MIAs accumulate during plant growth and development. The regulation of alkaloid biosynthesis is complex and still poorly understood. Lack of genetic tools is a major bottle-neck in identifying potential regulators involved in the pathways [31].

## Conclusions

In the present study, we utilized deep transcriptomics combined with metabolic profiling by LC-MS to illustrate the linkage between the expression of genes (TFs and candidate ATP-binding cassette (ABC) transporters) and regulation of TIA accumulation under the influence of ET and MeJA. Results showed that ET has a stronger effect on induction of TIAs synthesis at both transcriptional and metabolite level. By screening of the database, 23 ABC transporter partial sequences were identified in *C. roseus*. Furthermore, statistical analysis revealed that ERF and BIS1 formed a positive feedback loop connecting two ABC transporters respectively and were actively involved in TIAs responding to ET and MeJA in *C. roseus* (Fig. 8). Plant ABC transporters considerably contributed to membrane transportation of endogenous secondary metabolites in the plant body. However, there are still only limited examples of transportation studies on secondary metabolites in plant to date. *C. roseus* can be used as an appropriate model plant for secondary metabolism research and our data also provide important information for transportation mechanism in plants.

## Methods

### Plant material, growth conditions, and treatments

*Catharanthus* plants were grown in a growth chamber (ZPW-400, China), under a 12 h / 12 h light / dark photoperiod with irradiance of 450 μmol m^− 2^ s^− 1^. The temperature and relative humidity were controlled at 28 °C (day) / 25 °C (night) and 80% respectively as described previously.

Experiment 1: Three-month-old seedlings were transferred to Hoagland’s solution containing 30 μM of ethephon (ETH, Ethephon was used to release ethylene), 50 μM of MeJA and Hoagland’s solution without adding ETH or MeJA, which was used as a control. The seedlings were harvested for RNA-seq after 4 h treatment. Each treatment was performed with 3 replicates.

Experiment2: Three-month-old seedlings were transferred to Hoagland’s solution containing 30 μM of ethephon (ETH) or 50 μM of MeJA, while Hoagland’s solution without adding either ETH or MeJA was used as a control. Half seedlings were harvested for qRT-PCR after 4 h treatment (Roots, Stems, and Leaves) and seedlings remained were harvested for metabolites accumulation measurement after treated with 24 h (Roots, Stems, and Leaves). Each treatment was performed with 10 replicates.

### RNA preparation, sequencing and cDNA library construction

Treated seedling samples were immediately frozen in liquid nitrogen and stored at − 80 °C until further use. For Illumina sequencing, total *Catharanthus* RNA was isolated and then digested with DNase I follow manufacturer’s instructions (RNeasy Plant Mini Kit, Qiagen, Hong Kong). The concentration and quality of each sample were determined by using an Agilent 2100 Bioanalyzer RNA Nanochip. Only samples with a BioAnalyzer RNA Integrity Number (RIN) of 7.5 or greater were used for transcriptome sequencing. Poly(A) + RNA was purified at least 20 μg of total RNA by two rounds of selection using oligo (dT) attached to magnetic beads and a Dynabeads mRNA Purification kit (Invitrogen). Samples from control, ethylene-treated and MeJA-treated, with three biological replicates, were barcoded to adapt to multiplex of 9 samples per lane. 100 nt pair-end sequencing was performed by Illumina HiSeq2000. Raw reads from sequencing machines include adapter sequences and unknown or low-quality bases. These data will negatively affect following bioinformatics analysis. So low-quality raw reads were discarded sequentially: First, removing reads with adapters; second, removing reads with unknown nucleotides larger than 5%; third, removing low quality reads (The rate of reads with quality value ≤10 was more than 20%); fourth, obtaining the clean reads. All the downstream analyses were based on clean data with high quality. In these experiments, the same method published in Wang et al. [43] was applied.

### Transcriptome assembly and gene annotation

The read1 files from all samples were pooled into one big left.fq file, and read2 files into one big right.fq file. Transcriptome de novo assembly was carried out based on the left.fq and right.fq using Trinity (http://trinityrnaseq.sourceforge.net/) with min_kmer_cov set to 3 and all other parameters set to default [42]. Reads from all treatments were used to create a single reference assembly. The longest assembled sequences are called contigs. Then the reads are mapped back to contigs; with paired-end reads, it is able to detect contigs from the same transcript as well as the distances between these contigs. Finally, get sequences without Ns and cannot be extended on either end. Such sequences are defined as Unigenes. TGICL (http://sourceforge.net/projects/tgicl/files/tgicl%20v2.1/) is used to assemble all the unigenes from different samples to form a single set of non-redundant unigenes. Then do gene family clustering, the unigenes will be divided into two class. One is clusters, in which the prefix is CL and the cluster id is behind. In one cluster, there are several unigenes which similarity between them is more than 70%. And the other is singletons, which the prefix is Unigene. The assembled transcriptome file has been listed in Additional file 11: Table S9.

Redundancy removal tool Phrap (http://sourceforge.net/projects/tgicl/files/tgicl%20v2.1/) was used with the comprehensive non-redundant assembly. Then the reads from individual samples were mapped back to non-redundant comprehensive assembly using bowtie2 (http://bowtie-bio.sourceforge.net/bowtie2/index.shtml). The abundance and differential expression of the reads were measured by Cufflinks and Cuffdiff (http://cole-trapnell-lab.github.io/cufflinks/). All programs were performed using default parameters. We search All-Unigene sequences against protein databases (Nr, SwissProt, KEGG, COG) using blastx (e-value < 0.00001) With Nr annotation, Blast2GO software (https://www.blast2go.com/home) was used to define GO annotation by molecular function, cellular component, and biological process ontologies. After obtaining GO annotation for every unigene, WEGO software [28] was used to produce GO functional classification for all unigenes and to interpret the distribution of species’ gene functions at the macro level. GO enrichment analysis of DEGs was performed using hypergeometric test. GO terms with adjusted *P*-values of < 0.05 were defined as significantly enriched GO terms [43]. Pathway assignments were determined following the Kyoto Encyclopedia of Genes and Genomes (KEGG) pathway database [44]. Simple and reciprocal BLAST searches at an E-value cut-off of ≤1e-05 were conducted for identification of the best significant match [44]. Pathway enrichment analysis identifies significantly enriched metabolic pathways or signal transduction pathways in DEGs comparing with the whole genome background. The calculating formula of *p*-value is similar to that in GO enrichment analysis. We choose pathways with Qvalue ≤0.05 were significantly enriched in DEGs [44].

The RNA-seq raw data was submitted to NCBI under Sequence Read Archive (SRA) database (NCBI BioProject Accession: PRJNA358259, https://www.ncbi.nlm.nih.gov/bioproject?LinkName=biosample_bioproject&from_uid=6176568). Our assembled transcripts are deposited with the NCBI Transcriptome Shotgun Assembly (TSA) database under the accession: GFZE00000000. It can be accessible with the following link: https://www.ncbi.nlm.nih.gov/nuccore/GFZE00000000. The version described in this paper is the first version.

### Determination of alkaloid contents

Freshly treated plant seedlings (1.5 g) were frozen in liquid nitrogen and ground in 20 mL of methanol for metabolites extraction under low-frequency ultrasonication (250 W, 40 kHz) for 30 min. The methanol extracts were centrifuged at 8000 rpm for 10 min to separate tissue debris and the methanol extracts were dried in a vacuum dryer, and then re-dissolved in 1 mL of methanol for further analysis. The analyzes were separated using a COSMOSIL Packed Column C18 (5 μm, 4.6 × 250 mm) and detected by photodiode array and MS. The solvent systems for metabolites analysis were as follows: solvent A, 0.05 mol / L ammonium acetate; solvent B, acetonitrile. The following elution gradient was used: 47% A, 53% B (0–8 min); 47–42% A, 53–58% (10–23 min); 42–5% A, 58–95% B (23–33 min); and 5% A, 95% B, (33–45 min).

### Real-time PCR analysis

Total RNA was extracted by TRIzol reagent (Invitrogen) and used for cDNA synthesis (ReverTra Ace QPCR RT Kit, TOYOBO, Japan) after DNase I (TaKaRa, Japan) digestion. The expression of target genes as well as internal control, Ribosomal protein subunit 9 (*Rsp9*), was monitored by quantitative Real-Time PCR (qRT-PCR) using appropriate gene-specific primers (Additional file 12: Table S10). SYBR Premix Ex Taq system (TaKaRa, Japan) was used for gene expression analysis and parameters used for quantitative real-time PCR were 95 °C for 30 s, followed by 40 cycles of 94 °C for 30 s, 56 °C for 30 s and 72 °C for 30 s.

### Statistical analysis

The principal component analysis (PCA) was performed to evaluate variations of gene expression in response to ET and MeJA. The principal component scores explained the variation. The score of principal component “Q” is an indicator of a comprehensive analysis, scientific evaluation of objective phenomenon, which has no practical significance, and negative just means below the average [31, 45].

Results were subjected to analysis of variance (ANOVA) to determine the significant differences between treatments. When ANOVA was performed, Duncan’s significant difference post hoc tests were conducted to determine the differences between the individual treatments (SPSS 17. 0, SPSS Inc., USA). SPSS was also used to calculate the PCA.

## Additional files

Additional file 1: **Table S1**. Statistical summary of sequencing and assembly results. (XLS 21 kb). (XLS 21 kb)

Additional file 2: **Table S2**. Statistical summary of annotation results. (XLS 22 kb). (XLS 22 kb)

Additional file 3: **Table S3**. Statistical summary of DEGs. (XLS 412 kb). (XLS 411 kb)

Additional file 4: **Figure S1**. Gene Ontology classification. (DOC 338 KB) (DOCX 337 kb)

Additional file 5: **Table S4**. GO analysis of DEGs. (XLS 37 kb). (XLSX 36 kb)

Additional file 6: **Table S5**. KEGG analysis of DEGs. (XLS 62 kb). (XLS 78 kb)

Additional file 7: **Figure S2**. The effect of ET on metabolic pathway genes. (DOC 286 KB) (DOCX 285 kb)

Additional file 8: **Table S6**. GO analysis the genes of ET specific regulatory. (XLS 11 kb). (XLSX 10 kb)

Additional file 9: **Table S7**. The expression of TIA-relative genes. (TIA pathway genes, TFs, ABC transporters) in response to ET or MeJA in different tissues by qRT-PCR. (XLS 60 kb). (XLS 59 kb)

Additional file 10: **Table S8**. The FPKM value of 339 Candidate ABC Transporter Genes. (XLS 432 kb). (XLS 432 kb)

Additional file 11: **Table S9**. Assembled transcriptome file. (FASTA 100,416 MB). (FSA 100415 kb)

Additional file 12: **Table S10**. Primers used in qRT-PCR for validation of DEGs. (XLS 17 kb). (XLSX 16 kb)

## Abbreviations

10-HGO: 10-hydroxygeranioloxidoreductase
7DLS: 7-deoxyloganetic acid synthase
ABC: ATP-binding cassette
ANOVA: Analysis of variance
AP2/ERF: APETALA2/ethylene response factor
AS: Anthranilate synthase
BIS: bHLH iridoid synthesis
BLASTX: Basic local alignment search tool x
BPF: Parsley box P-binding factor
BZIP: Basic leucine-zipper
CBF: C-repeat binding factor
CDR: CoA-disulfide reductase
CK: Control
CMK: 4-diphosphocytidyl-2-C-methyl-D-erythritol kinase
CMS: 4-diphosphocytidyl-2-C-methyl-D-erythritol synthase
COG: Clusters of orthologous groups of proteins
D4H: Desacetoxyvindoline − 4-hydroxylase
DAT: 4-O-deacetylvindoline4-O-acetyl transferase
DEGs: Differentially expressed genes
DL7H: 7-deoxyloganic acid 7-hydroxylase
DLGT: 7-deoxyloganetic acid glucosyltransferase
DXR: 1-deoxy-D-xylulose-5-phosphate reductoisomerase
DXS: 1-deoxy-D-xylulose-5-phosphate synthase
ERF: Ethylene responsive factor
ET: Ethylene
FDR: False discovery rate
FPKMs: Fragment per kilobase of exon model per million mapped reads
G10H: Geraniol 10-hydroxylase
GA: Gibberellic acid
GBF: G-box--binding factors
GFP: Green fluorescent protein
GO: Gene ontology
HDR: 1-hydroxy-2-methyl-2-butenyl 4-diphosphate reductase
HDS: 1-hydroxy-2-methyl-2-butenyl 4-diphosphate synthase
HPLC: High performance liquid chromatography
IRS: Iridoid synthase
JAZ: Jasmonate ZIM domain-containing protein
KEGG: Kyoto encyclopedia of genes and genomes
LAMT: Loganic acid O-methyltransferase
LC-MS: Liquid chromatograph-mass spectrometer
LUC: Luciferase
MAPK: Mitogen activated protein kinase
MECS: 2-C-methyl-D-erythritol 2, 4-cyclodiphosphate synthase
MeJA: Methyl jasmonate
MIA: Monoterpenoid indole alkaloids
MVA: Mevalonate
MVK: Mevalonate kinase
MYC: Myelocytomatosis oncogene
RPS: Ribosomal protein subunit
SA: Salicylic acid
SGD: Strictosidine b-D-glucosidase
SLS: Secologanin synthase
SRA: Sequence read archive
STR: Strictosidine synthase
T16H: Tabersonine-16-hydroxylase
TDC: Tryptophan decarboxylase
TIA: Terpenoid indole alkaloid
TPT: Triose-phosphate transporter-like protein
ZCT: Zinc finger *Catharanthus* transcription factor
