# Supplementary material for: Transcriptomics comparison reveals the diversity of ethylene and methyl-jasmonate in roles of TIA metabolism in Catharanthus roseus
Source: BMC Genomics. 2018 Jul 2;19:508. doi: 10.1186/s12864-018-4879-3 (PMC6029152; doi:10.1186/s12864-018-4879-3)
Supplement: Supplementary file 4 — Figure S1. Gene Ontology classification. (DOC 338 KB) (DOCX 337 kb) [file 12864_2018_4879_MOESM4_ESM.docx]

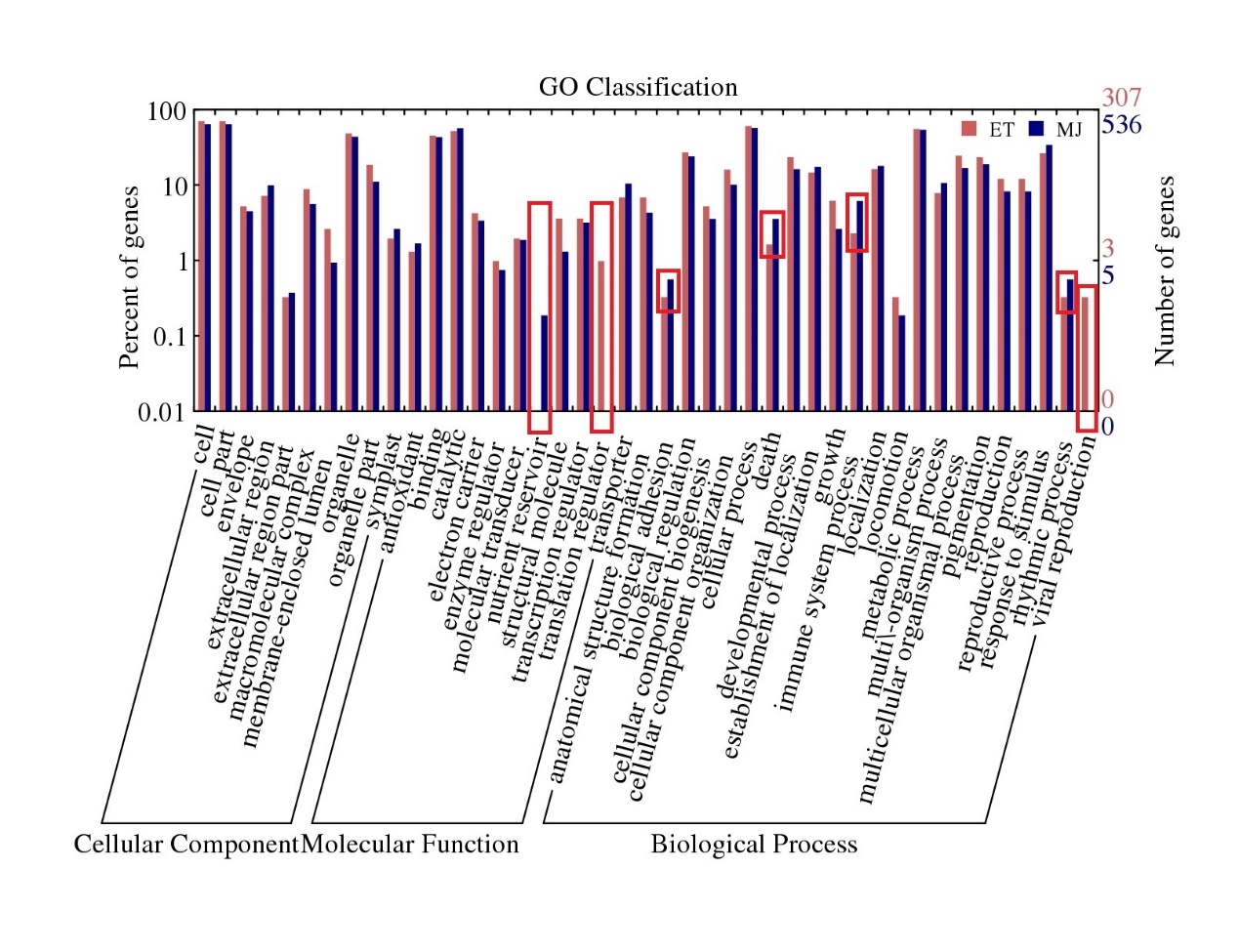


**Additional file Figure S1 Gene Ontology classification**

The unigenes are summarized into three main categories: biological process, cellular location, and molecular function. The left Y-axis represents the percentage of a specific category of genes in each main category, and the right Y-axis represents the number of genes in a category.
