# Supplementary material for: Transcriptomics comparison reveals the diversity of ethylene and methyl-jasmonate in roles of TIA metabolism in Catharanthus roseus
Source: BMC Genomics. 2018 Jul 2;19:508. doi: 10.1186/s12864-018-4879-3 (PMC6029152; doi:10.1186/s12864-018-4879-3)
Supplement: Supplementary file 7 — Figure S2. The effect of ET on metabolic pathway genes. (DOC 286 KB) (DOCX 285 kb) [file 12864_2018_4879_MOESM7_ESM.docx]

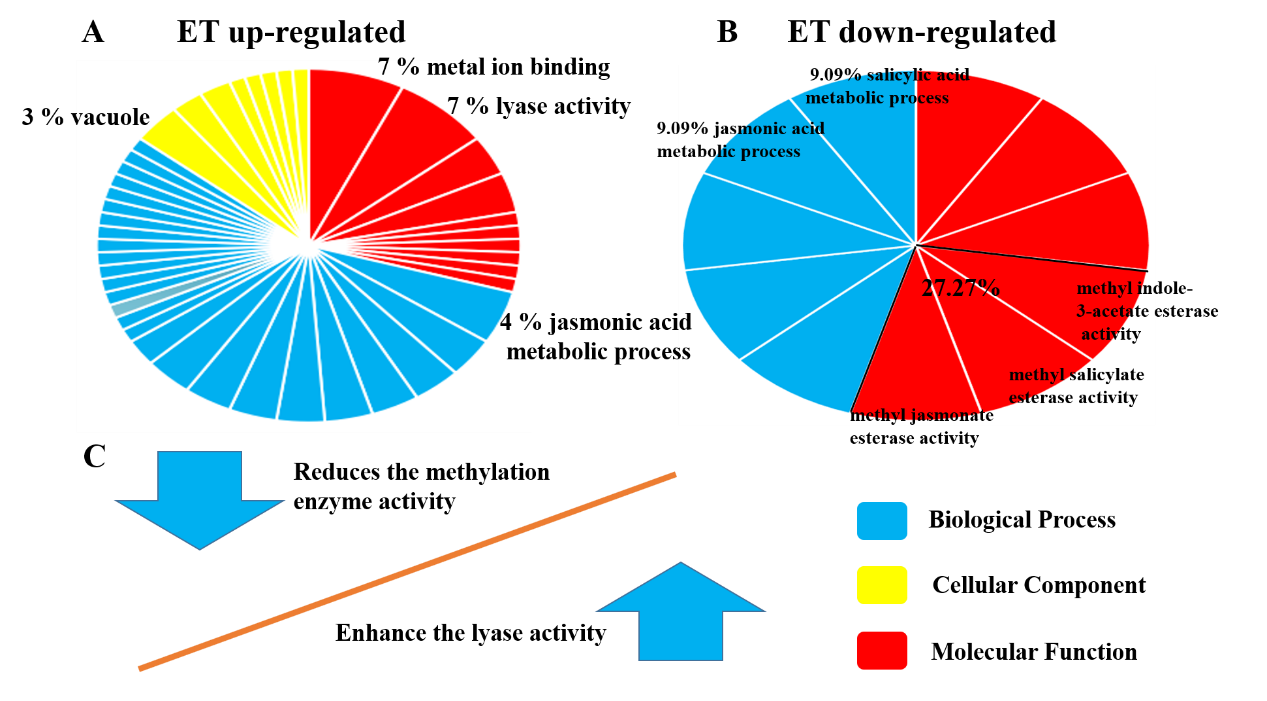


**Additional file Figure S2 The effect of ET on metabolic pathway genes**

The 197 metabolic pathway genes were further analysis by GO annotation. A. The 33 up-regulated genes in response to ET. B. The 35 down-regulated genes in response to ET. The red part denoted molecular function, the yellow part denoted cellular component, and the blue part denoted biological process. C. The effect of ET on enzyme activity.
